# Lymphatic therapies open the valve in Marfan syndrome

**DOI:** 10.1172/JCI209169

**Published:** 2026-08-03

**Authors:** Yanna Tian, Kathleen M. Caron

**Affiliations:** Department of Cell Biology and Physiology, The University of North Carolina at Chapel Hill, Chapel Hill, North Carolina, USA.

## Abstract

Myxomatous degeneration of the mitral valve (MDMV) is a common cardiovascular manifestation of Marfan syndrome (MFS), yet the role of lymphatic vessels in the disease progression remains unknown. In this Commentary, we discuss the study by Tan, Kume, and colleagues, which identifies defective lymphangiogenesis as a previously unrecognized driver of MDMV. Their work demonstrates that impaired lymphatic development and drainage promote valve inflammation through reduced ZFP36-mediated antiinflammatory signaling, whereas restoration of lymphatic function or pharmacological activation of ZFP36 with the FDA-approved drug FTY720 ameliorates disease progression. These findings establish lymphatic vessels as critical regulators of mitral valve homeostasis and support exploration of lymphatic-targeted therapeutic opportunities for MFS-associated valvular disease.

## Marfan syndrome: causes, symptoms, and treatments

Marfan syndrome (MFS) was named after Antoine Marfan, who first reported a child with distinguishing skeletal features in 1896 and later reported 150 additional cases. In 1955, Victor McKusick described the cardiovascular features of MFS using echocardiography (1). MFS is a multisystem connective tissue disorder defined by ocular abnormalities (lens dislocation and myopia), skeletal features (dolichostenomelia and joint laxity), and, most importantly, cardiovascular pathology, including aortic root dilatation, dissection, mitral valve prolapse (MVP), and valvular insufficiency (2).

MFS is most commonly caused by pathogenic variants in the *FBN1* gene, which encodes Fibrillin-1 (3, 4). Approximately 75% of affected individuals inherit the disorder in an autosomal manner, while 25% of cases arise from de novo mutations. Nearly 3,000 rare *FBN1* variants have been identified, contributing to phenotypic variability.

FBN1 is a large ECM glycoprotein that assembles into microfibrils, providing structural integrity, elasticity, and resilience to tissues such as arteries, cardiac valves, lung, and skin. Beyond structural support, FBN1-containing microfibrils regulate growth factor signaling by sequestering TGF-β family members, including TGF-β1 and bone morphogenetic proteins. Pathogenic variants in *FBN1* disrupt microfibril assembly and growth factor sequestration, leading to excessive TGF-β accumulation, a primary driver of aortic disease in patients with MFS (4, 5).

### Pathophysiology of myxomatous degeneration of the mitral valve.

Myxomatous degeneration of the mitral valve (MDMV) and MVP are common manifestations of MFS, affecting approximately 16.1% of patients with MFS (6). Disease progression is characterized by thickened, elongated, and mechanically weakened valve leaflets due to ECM remodeling, often resulting in mitral regurgitation. According to the Ghent II criteria, MVP is one of the clinical features for the diagnosis of MFS (3). Although the prevalence and severity vary, valvular disease is especially severe in neonatal MFS, where MVP and regurgitation are major causes of morbidity and mortality (7, 8). Like aortic pathology, MDMV is largely driven by *FBN1* mutations, with increased TGF-β/SMAD signaling observed in both human tissues and animal models (9–13). Recent studies have expanded this paradigm, identifying integrin-mediated mTOR signaling as a contributor to TGF-β overactivity due to *FBN1* mutations (14). Additional mechanisms include altered mechanotransduction and inflammatory signaling. Reduced endothelial KLF2/4 expression, increased SMAD signaling, and enhanced monocyte recruitment have been observed in both experimental models and MDMVs (15).

Immune cell infiltration has emerged as another important contributor to the progression of MDMV in MFS. Notably, increased accumulation of proinflammatory macrophages has been demonstrated in myxomatous valves from *FBN1* mutant mice, sheep, pigs, and human patients (16). These macrophages are derived predominantly from CCR2^+^ classical monocytes (16), implicating monocyte recruitment and inflammation as key drivers of disease progression. Collectively, dysregulated ECM signaling, impaired mechanosensing, and chronic inflammation cooperate to drive mitral valve degeneration. In this issue of the *JCI*, Tan, Kume, and colleagues extended this framework by identifying a previously unrecognized link between disrupted lymphangiogenesis and MDMV progression in *Fbn1* mutant mice (17).

## Lymphatic vessels in the mitral valve

Lymphatic vessels are essential for maintaining fluid homeostasis, mediating immune cell trafficking, and resolving inflammation. In the heart, the lymphatic network plays an increasingly recognized role in tissue remodeling and immune regulation under both physiological and pathological conditions. Evidence for lymphatic involvement in valvular disease has been reported in rheumatic heart valve disease and infective endocarditis, where lymphatic vessels are present within diseased leaflets (18, 19). Previous studies show lymphatics localized to ECM-rich regions, with increased abundance in inflammatory conditions such as infective endocarditis (18). Lymphangiogenesis is also associated with cardiac valvular disorders, such as rheumatic heart valve disease (19). Other recent work by the Kume group suggests that lymphatic vessels help maintain mitral valve structure by regulating interstitial fluid balance and ECM turnover (20). Despite these observations, the developmental origin and functional significance of valve lymphatics remain poorly understood. In particular, it has been unclear whether FBN1 mutations impair lymphatic formation and function or whether restoring lymphatic activity could provide therapeutic benefit.

As mentioned above, previous studies by the Kume group demonstrated lymphatic vessels beneath valvular endothelial cells on the atrial side of mouse mitral valves (20). In the current study, Tan and colleagues examined lymphatic development in WT and *Fbn1* heterozygous (*Fbn1^C1039G/+^*) mice. Whole-mount staining revealed that VEGFR3^+^ lymphatic vessels expanded postnatally into the mitral annulus at P3, sprouting from regions above the anterior commissure. Lymphatic growth continued into both leaflets through P14 in WT mice. In contrast, *Fbn1* mutant mice exhibited reduced lymphatic proliferation, with decreased vessel density and branching at P14, along with abnormal valve enlargement. These defects were likely driven by elevated TGF-β signaling suppressing lymphangiogenesis. Structural abnormalities were also observed in lymphatic endothelial cell junctions. Whereas normal lymphatic vessels displayed discontinuous button-like junctions in capillaries and continuous zipper-like junctions in collecting vessels, mutant mice exhibited predominantly button-like junctions throughout. These abnormalities impaired lymphatic drainage, leading to fluid accumulation and increased valve thickness in MFS mice.

To test causality, the authors used VEGF-C156S, a specific VEGFR3 ligand, to promote lymphangiogenesis and rescue MDMV phenotypes in *Fbn1* mutant mice. Treatment restored VEGFC/VEGFR3 signaling via increased ERK1/2 and Akt phosphorylation, increased lymphatic vessel density, and significantly reduced mitral valve thickening. These findings establish defective lymphangiogenesis as a key contributor to MDMV pathogenesis, suggesting that restoring lymphatic function can ameliorate MDMV in MFS.

## FDA-approved FTY720: a potential therapy to prevent MDMV

Tan et al. also observed that lymphatic dysfunction in *Fbn1* mutant valves was associated with disrupted endothelial organization and increased macrophage infiltration. Reanalysis of scRNA-seq data identified ZFP36 as a key regulator of MDMV progression.

ZFP36 (tristetraprolin) is an antiinflammatory protein that promotes degradation of proinflammatory mRNAs by binding AU-rich elements in their 3′ untranslated regions. In *Fbn1* mutant valves, ZFP36 expression was reduced across multiple cell types, including endothelial cells, macrophages, and DCs, indicating loss of antiinflammatory regulation. Endothelium-specific deletion of *Zfp36* (*Cdh5-Cre^ERT2^ Zfp36^fl/fl^* mice) resulted in increased immune cell infiltration and valve thickening, recapitulating MDMV phenotypes.

To restore ZFP36 function, the authors used FTY720 (fingolimod), an FDA-approved drug for multiple sclerosis (21). Although best known as a modulator of sphingosine-1-phosphate receptor (S1PR) signaling, FTY720 activates protein phosphatase 2A, which enhances ZFP36 activity by promoting its dephosphorylation and stabilization (22–24). Daily oral administration of FTY720 from P1 to P59 significantly reduced CD45^+^ immune cell infiltration in *Fbn1* mutant valves in mice. Mitral valve abnormalities, including leaflet elongation and thickening, were attenuated, and cardiac function was improved, with restored ejection fraction and fractional shortening, by FTY720 administration in *Fbn1* mutant mice.

FTY720 also influenced lymphatic vessels in unexpected ways. While FTY720 reduced lymphatic density in WT valves, it did not alter the lymphatic density in *Fbn1* mutant valves. FTY720 binds S1PRs, promoting receptor internalization and degradation, and decreasing S1PR immunostaining in lymphatic endothelial cells of WT mice. In a mechanism that remains yet unclear, the *Fbn1* mutant mitral valves did not show a decrease in S1PR levels with FTY720 treatment. Interestingly, however, in *Fbn1* mutant mice, FTY720 alleviated MDMV phenotypes by promoting continuous zipper-like junctions in the lymphatic endothelial cells, reducing the number of reticular adherens junctions in valvular endothelial cells, enhancing lymphatic drainage, and reducing immune cell accumulation. These findings demonstrate that improving lymphatic function, even without increasing vessel number, can mitigate disease.

## Conclusions and future directions

**Figure 1:**
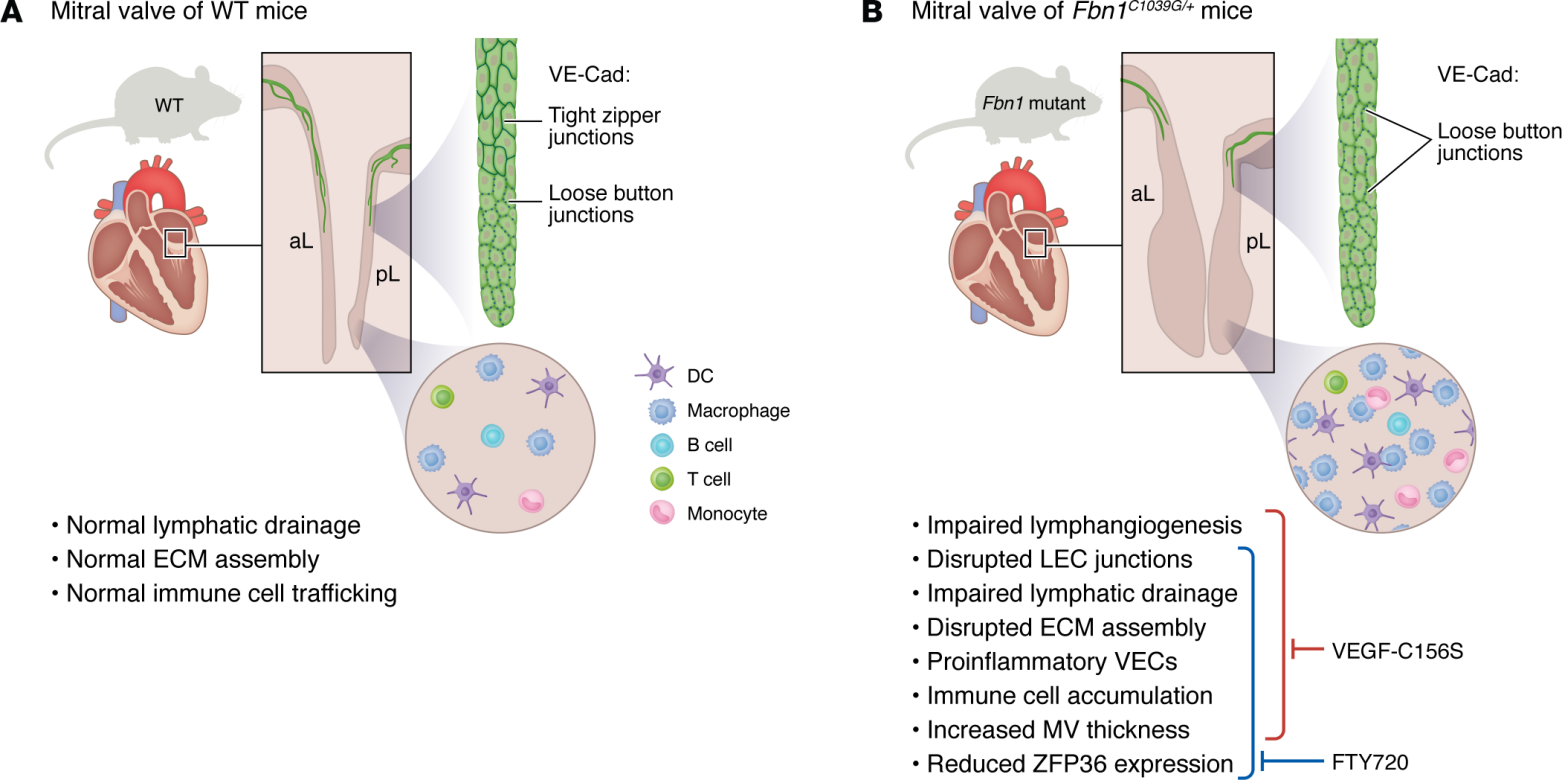
Implicating defective lymphatic vessel development in MFS-associated valvular disease. Tan et al. (17) compared the development of VEGFR3^+^ lymphatic vessels (LVs) in the mitral valve (MV) of WT and *Fbn^C1039G/+^* mice, a model of MFS. (**A**) In WT mice, LVs emerged from the ventricle and septum and progressed from the anterior triangle (appearance at P0) to the anterior leaflet (aL) (invasion by P7), with posterior leaflet (pL) branching by P14. Normal lymphatic endothelial cells (LECs) displayed discontinuous VE-cadherin junctions in capillaries and continuous zipper junctions in collecting vessels, structures that are essential for maintaining LV function and MV morphology. (**B**) *Fbn^C1039G/+^* MVs exhibited disrupted VE-cadherin junctions, which corresponded with reduced LV density, impaired lymphatic drainage, and immune cell accumulation, leading to MDMV. VEGF-C156S restored VEGFC/VEGFR3 signaling, promoted lymphangiogenic extension, and corrected LV and MDMV defects. Mutant MVs also showed reduced ZFP36, and FDA-approved FTY720 enhanced ZFP36 activity via PP2A, stabilizing VE-cadherin junctions, improving lymphatic function, and mitigating MDMV. VEC, valvular endothelial cell.

This study advances our understanding of MDMV in MFS by identifying lymphatic dysfunction and impaired antiinflammatory regulation as central contributors. Restoration of lymphangiogenesis using VEGF-C156S and pharmacological activation of ZFP36 using FTY720 both significantly improved valve pathology in *Fbn1* mutant mice (Figure 1). Current MFS management relies primarily on surgical repair and pharmacologic treatments such as beta blockers and angiotensin receptor blockers, including losartan. Losartan inhibits TGF-β signaling and has been shown to prevent aortic aneurysm formation in *Fbn1^C1041G/+^* mice, with efficacy comparable with TGF-β–neutralizing antibodies and superior to β-blockade alone (25). However, the clinical efficacy of the add-on effect of losartan with beta blockers is inconsistent (26). The findings presented here highlight therapeutic avenues beyond conventional treatments. Targeting lymphatic vessel growth and function, along with modulation of inflammatory pathways via ZFP36, offers promising strategies for preventing and treating mitral valve disease in MFS. Additional studies are needed to determine whether the pathways identified by Tan et al. and lymphatic-targeted therapies can be utilized to alter the clinical course of mitral valvular disease in patients with MFS.

Author contributions: KMC and YT drafted, edited, and reviewed the manuscript. YT generated the figure using an academic institutional BioRender license (CU29KSQ8UB).

## Conflict of interest

The authors have declared that no conflict of interest exists.

## Funding support

This work is the result of NIH funding, in whole or in part, and is subject to the NIH Public Access Policy. Through acceptance of this federal funding, the NIH has been given a right to make the work publicly available in PubMed Central.

- NIH grant from the Eunice Kennedy Shriver National Institute of Child Health and Human Development (HD060860).
- Advanced Research Projects Agency for Health award ICHUB-24-101-1409 to KMC.
- American Heart Association postdoctoral fellowship 24POST1188946.
- National Heart, Lung, and Blood Institute K99/R00 award 1K99HL186015-01 to YT.

## Version 1 08/03/2026

Electronic publication
